# The Teeth

**Published:** 1884-11

**Authors:** 


### THE TEETH.

WE don't like to allow an issue of our magazine to go to the world unless it contains a few words of counsel and warning upon the necessity of taking care of ths teeth. Not a day passes in which we are not forcibly reminded of the terrible thoughtlessness of the human family in this important particular.

More than half the people whom we meet have a bad-smelling mouth, and teeth the beauty of which has been seriously injured by want of care. It is no use to say "Why, teeth decay naturally," for teeth never decay naturally. Decay in teeth is simply solution, and nature does not make teeth for the simple purpose of dissolving them in the juices of the mouth. The talk about parasites and destructive microscopical insects who feed on the teeth, and are never so happy as when lunching on a molar, is all very well, but is in the main fallacious; for there are no animals about the teeth which require a weapon more formidable for their destruction than a good firm tooth-brush, dipped in water, and charged with a little soap or chalk. If parents will provide their children with hard, flexible brushes, and see that they are used invariably after eating, they will do them a more valuable service than by leaving them many thousand dollars in money. Not only do neglected and decaying teeth make the individual offensive to all cleanly persons who come in contact with him, but they are frequently the cause of the most painful, and fatal diseases. It is safe to say, that the most excruciating cases of facial neualgia can always be traced to decaying teeth. The little nerves contained in the bony substance of the teeth may form the seeds that impart disease to the whole nervous system.

The decaying matter from a single ulcerated tooth, may be sufficient to poison the blood, beyond recovery. Every good dentist is a philanthropist, and should be well patronized.

**Housekeepers** should recollect that it is the penny saved, more than the penny earned, that enriches; it is the sheet, or blanket, or garment darned when the first thread breaks that wears the longest; it is the dampers closed when the cooking is done that stops some unnecessary dollars from dropping into the coal bin; it is the lamp or gas turned low when not in use that gives you pin money for the month; it is taking care to see that the coffee is browned instead of being burned or merely dried, that it is ground fine instead of being coarsely cracked, which makes three spoonfuls go as far as a cupful; that, in short, it is eternal vigilance in little things which makes the difference between the thoughtless poor and the forehanded middle-class.

